# The effect of transcranial direct current stimulation (tDCS) combined with cognitive training on EEG spectral power in adolescent boys with ADHD: A double-blind, randomized, sham-controlled trial

**DOI:** 10.1016/j.ibneur.2021.12.005

**Published:** 2021-12-24

**Authors:** Samuel J. Westwood, Natali Bozhilova, Marion Criaud, Sheut-Ling Lam, Steve Lukito, Sophie Wallace-Hanlon, Olivia S. Kowalczyk, Afroditi Kostara, Joseph Mathew, Bruce E. Wexler, Roi Cohen Kadosh, Philip Asherson, Katya Rubia

**Affiliations:** a Department of Child & Adolescent Psychiatry, Institute of Psychiatry, Psychology and Neuroscience, King’s College London, London SE5 8AB, UK; b School of Psychology, University of Wolverhampton, Wolverhampton WV1 1LY UK; c Department of Psychology, School of Social Science, University of Westminster, London W1W 6UW, UK; d Social Genetic & Developmental Psychiatry Centre, Institute of Psychiatry, Psychology and Neuroscience, King’s College London, London SE5 8AB, UK; e School of Psychology, University of Surrey, Guildford GU2 7XH, UK; f Department of Neuroimaging, Institute of Psychiatry, Psychology and Neuroscience, King’s College London, London SE5 8AB, UK; g Department of Psychiatry, Yale University School of Medicine, 06520–8096, USA

**Keywords:** ADHD, TDCS, Treatment, EEG

## Abstract

Transcranial direct current stimulation (tDCS) is a possible alternative to psychostimulants in Attention-Deficit/Hyperactivity Disorder (ADHD), but its mechanisms of action in children and adolescents with ADHD are poorly understood. We conducted the first 15-session, sham-controlled study of anodal tDCS over right inferior frontal cortex (rIFC) combined with cognitive training (CT) in 50 children/adolescents with ADHD. We investigated the mechanisms of action on resting and Go/No-Go Task-based QEEG measures in a subgroup of 23 participants with ADHD (n, sham = 10; anodal tDCS = 13). We failed to find a significant sham versus anodal tDCS group differences in QEEG spectral power during rest and Go/No-Go Task performance, a correlation between QEEG and Go/No-Go Task performance, and changes in clinical and cognitive measures. These findings extend the non-significant clinical and cognitive effects in our sample of 50 children/adolescents with ADHD. Given that the subgroup of 23 participants would have been underpowered, the interpretation of our findings is limited and should be used as a foundation for future investigations. Larger, adequately powered randomized controlled trials should explore different protocols titrated to the individual and using comprehensive measures to assess cognitive, clinical, and neural effects of tDCS and its underlying mechanisms of action in ADHD.

## Highlights

• The mechanism of action of tDCS in ADHD are poorly understood.
• We combined 15-session anodal tDCS over the rIFC with cognitive training in ADHD children.
• EEG was measured at rest and during a cognitive control task.
• Real vs sham tDCS showed no significant effect on EEG at rest or during Go/No-Go task.
• Real versus sham tDCS showed no significant cognitive or symptom improvements.

## 1 Introduction

Attention-Deficit/Hyperactivity Disorder (ADHD) is neurodevelopmental disorder marked by age-inappropriate, and impairing symptoms of inattention and/or impulsivity-hyperactivity (APA, 2013). ADHD is also associated with deficits in executive functions (EF), including motor and interference inhibition, sustained attention, switching, working memory (WM), and timing (Rubia, 2018), underpinned by neurofunctional abnormalities in inferior and dorsolateral fronto-striatal and fronto-cerebellar regions based on functional magnetic resonance imaging (fMRI) meta-analyses(Hart et al., 2014; Hart et al., 2012; Lukito et al., 2020; Norman et al., 2016). This atypical frontal brain activity in ADHD is further related to an increase in slow-wave cortical activity, as reflected in excessively increased electroencephalographic (EEG) power in theta and delta over frontal and central brain regions in both adults and children with ADHD (Kiiski et al., 2020, McVoy et al., 2019).

The gold-standard treatment for ADHD are psychostimulants, which improve ADHD symptoms in roughly 70% of individuals with ADHD(UK, 2018). However, psychostimulants have been associated with side-effects (Faraone et al., 2015), poor adherence in adolescence (Cortese et al., 2018, Cunill et al., 2016), while evidence of longer-term efficacy is limited (Cortese et al., 2018, Swanson et al., 2018), possibly due to brain adaptation (Fusar-Poli et al., 2012). Meta-analyses of alternative treatments, such as behavioral therapies, cognitive training (CT), or dietary interventions (Catalá-López et al., 2017), result in small to moderate improvement in ADHD symptoms.

A promising neurotherapeutic alternative is transcranial direct current stimulation (tDCS), which can potentially modulate key dysfunctional brain regions associated with ADHD with longer-term neuroplastic effects that drugs cannot offer (Cinel et al., 2019, Rubia, 2018, Sierawska et al., 2019, Westwood et al., 2020). TDCS involves applying a weak direct electrical current via two electrodes (one anode, one cathode) placed on the scalp, which modulate the excitability of underlying brain regions via polarity-dependent, subthreshold shifts in resting membrane potentials. The net increase or decrease in neuronal excitability (under the anode or cathode, respectively) can modulate neuronal network activity (Liu et al., 2018), with these effects persisting after stimulation due to practice-dependent changes in synaptic plasticity, mediated by GABA, glutamate (Filmer et al., 2019, Fonteneau et al., 2018, Kuo et al., 2008, Monte‐Silva et al., 2010). Furthermore, unlike other forms of non-invasive brain stimulation, such as transcranial magnetic stimulation, tDCS is cheaper, easier to use, and well tolerated with minimal side effects (Bikson et al., 2016).

Systematic reviews and meta-analyses of tDCS studies in ADHD suggest limited evidence of clinical or cognitive improvement with tDCS (Brauer et al., 2021, Cosmo et al., 2020, Salehinejad et al., 2019). However, the majority of studies applied 1–5 tDCS sessions over mainly left dorsolateral prefrontal cortex (DLPFC), and only one study applied 5 sessions over the right inferior frontal cortex (IFC) (Breitling-Ziegler et al., 2021). We conducted the only randomized sham-controlled study so far that stimulated the IFC, a region most consistently shown to be under-functioning in ADHD(Lukito et al., 2020; Norman et al., 2016), over more than 5 sessions (i.e., 15 sessions) combined with cognitive training (CT) in 50 boys aged 10- to 18-years with ADHD to potentiate cognitive and clinical effects(Jones et al., 2015; Kekic et al., 2016; Moffa et al., 2018; Ruf et al., 2017; Stephens and Berryhill, 2016; Tortella et al., 2015). While both groups improved, we failed to show group differences in improvements in clinical symptoms or in cognitive performance (including motor and interference inhibition, sustained attention and vigilance, time estimation, visuo-spatial WM, and cognitive flexibility) immediately after treatment or at a 6-month follow-up (Westwood et al., 2021).

Hardly anything is known about the neurophysiological substrates of tDCS effects in ADHD, with only three studies investigating these. In a double-blind, crossover RCT study with 10 adolescents with ADHD, single-session conventional anodal tDCS, anodal High Definition (HD)-tDCS or sham tDCS over rIFC led to enhanced N2 and P3 amplitude during an n-back WM task compared to sham (Breitling et al., 2020). In a double-blind RCT, 37 adults with ADHD received single sessions of sham or anodal tDCS over left or right DLPFC in a crossover design, with 18 participants performing an Eriksen Flanker task and 19 performing a Stop Signal task (Dubreuil-Vall et al., 2020). Participants showed reduced reaction times following left DLPFC and increased P3 amplitude following right and left DLPFC compared to sham in the Eriksen flanker task only, suggesting evidence of improved interference, but not response inhibition (Dubreuil-Vall et al., 2020). Using a functional cortical network (FCN) analysis on EEG activity, 50 adults with ADHD in a sham-control RCT showed increased functional brain connectivity within the stimulated and correlated areas after single-session anodal tDCS over the left DLPFC compared to baseline but not sham (Cosmo et al., 2015), thus we cannot rule out whether this improvement from baseline was incidental or a result of anodal tDCS specifically (Cosmo et al., 2015). Given the scarcity of neurophysiological investigations in ADHD following tDCS, the present study investigated the mechanism of action of tDCS using EEG spectral power during rest and during a Go/No-Go motor inhibition task.

Compared to event-related potentials (ERPs), EEG spectral power has been a preferred measure of treatment/stimulation response in both clinical and non-clinical studies. Findings in healthy adults on the effects of tDCS on spectral power are mixed. In one RCT, single-session anodal tDCS over the rIFC led to a reduction in absolute theta power at rest and improved inhibitory performance compared to sham (Jacobson et al., 2012), suggesting that theta power might be the neural signature of successful post-treatment inhibition (Jacobson et al., 2012). Further, compared to sham, RCTs with single-session anodal tDCS has also been shown to reduce frontro-central theta when stimulating left DlPFC (Mancini et al., 2016) or enhanced theta-gamma coupling when stimulating right PFC (Jones et al., 2020). EEG mean frequency was also found to be significantly reduced after both anodal and sham tDCS over the left DLPFC, although the effects were smaller for sham tDCS (Boonstra et al., 2016). By contrast, more recent RCTs found no effects on both rest- and task-based EEG power spectrum following anodal tDCS (Hill et al., 2019, Holgado et al., 2019, Miller et al., 2015), supporting a quantitative review that indicated little-to-no reliable neural effects of tDCS beyond motor evoked potentials (MEP)(Horvath et al., 2015b), although these findings might be due to small sample sizes and diverse methodology (e.g., differential measures and protocols) leading to discrepancy across non-clinical studies (Kim et al., 2018).

To our knowledge, this is the first study to examine the neuromodulatory effects of multi-session anodal tDCS combined with cognitive training over the rIFC on EEG spectral power in children and adolescents with ADHD. Based on aforementioned findings in healthy adults, we hypothesized that 15 sessions of anodal versus sham tDCS over the rIFC combined with multi-EF training would lead to a decrease during rest and an increase during task performance in absolute theta power. We also hypothesized that this effect would be associated with improved performance during a motor response inhibition Go/No-Go task.

## 2 Material and methods

### 2.1 Design

In a double-blind, sham-controlled, parallel RCT (ISRCTN: 48265228), 50 boys with ADHD received 15 sessions of anodal or sham tDCS over the rIFC combined with multi-EF training over 3 weeks (Westwood et al., 2021). We measured ADHD symptoms and related behaviors, ADHD-relevant EF (including motor and interference inhibition, sustained attention & vigilance, time estimation, working memory, and cognitive flexibility), safety, and EEG outcome measures at baseline, post-treatment, and 6-month follow-up. A more detailed experimental design can be found elsewhere (Westwood et al., 2021). Briefly, across 15 consecutive weekdays, participants received 20-minutes of 1 mA anodal or sham tDCS over the rIFC (F8; cathode over right supra-orbital area, Fp1) while playing cognitive training games composed of ACTIVATE™ games (to train visuo-spatial WM, selective attention, switching, and inhibition) and a training version of the Stop Task (to train motor inhibition)(Westwood et al., 2021) Sham tDCS was identical to anodal tDCS except the current was administered for 60 s (i.e., a 30 s fade-in/fade-out)(Westwood et al., 2021).

### 2.2 Participants

For the purposes of this paper, only participants that completed baseline and post-treatment EEG recordings were included. This is because, of the 50 participants, 21 had no EEG data recorded at all, while only 16 participants had EEG data recorded at the 6-month follow-up, which was too few for data analysis. Thus, this left 13 participants in the anodal and 16 in the sham tDCS group with baseline and post-treatment EEG eligible for data analysis.

Twenty-nine male participants (10- to 18-years) had a clinical DSM-5 diagnosis of ADHD assessed by an experienced child psychiatrist and confirmed using the Schedule of Affective Disorders and Schizophrenia for School‐Age Children‐Present and Lifetime version (K‐SADS‐PL)(Westwood et al., 2021). Participants also had to score above cut-off on Conners 3rd Edition–Parent Rating Scale (Conners 3-P, cut-off t-score > 60)(Westwood et al., 2021), and were screened for Autism Spectrum Disorders (ASD) using the parent-rated Social Communication Questionnaire (SCQ, cut-off > 17)(Westwood et al., 2021) and the pro-social scale of the Strengths & Difficulties Questionnaire (SDQ, cut-off < 5)(Westwood et al., 2021). Participants were excluded with IQs < 80 (Wechsler abbreviated scale of intelligence, WASI-I)(Westwood et al., 2021), a history of alcohol or substance abuse, neurological illness, comorbid major psychiatric disorders (except Conduct Disorder [CD]/Oppositional Defiant Disorder [ODD]); and tDCS contraindications. Consent was obtained from either the legal caregiver for participants under 16-years or from participants over 16-years (Figure 1). Participants received £540 for participating and were reimbursed for travel expenses.

Baseline assessment was scheduled at least two weeks after medication titration. Eighteen participants received stable ADHD medications (non-psychostimulants: 4; psychostimulants: 14; between 3-weeks and 9-years). To minimize the risk that psychostimulants might mask the effect of stimulation, participants on psychostimulants were asked to abstain for at least 24 h before each assessment session. Of the 12 participants who abstained, 5 chose to abstain throughout the trial period (>24 h before baseline until after post-assessment), while 7 abstained 24 h before each the baseline and post-assessment only (see Table 1).

**Table 1:** Baseline demographic, medication, clinical, and cognitive measures; the number of tDCS and CT sessions; and the time spent playing each CT game in the sham and anodal tDCS groups.

	Anodal tDCS N = 10		Sham tDCS N = 13		Independent *t*-test	
Demographics	Mean	SD	Mean	SD	t (2, 21)	p
*Age (months)*	154.30	23.03	174.07	22.38	-2.07	0.05
*IQ (WASI-II)*	104.50	15.40	106.77	13.73	-0.37	0.71
*Years in education*	8.40	2.01	10.15	1.82	-2.19	0.04
*SCQ*	9.60	5.582	8.62	6.826	0.37	0.72
*SDQ (Prosocial)*	7.00	2.055	6.31	1.974	0.82	0.42
*Kiddie-SADS (ADHD Symptoms)*						
Combined	12.40	3.44	12.15	2.70	0.19	0.85
Inattention	7.60	1.27	7.69	0.95	-0.20	0.84
Hyperactivity/impulsivity	4.80	2.70	4.46	2.47	0.31	0.76
**Clinical Measures & Side Effects**						
*ADHD-Rating Scale*						
Total Score	44.10	7.520	38.31	6.033	2.05	0.05
Inattention Subscale	24.60	2.413	21.46	3.799	2.28	0.03
Imp/Hyp Subscale	19.50	6.133	16.85	3.738	1.29	0.21
*Conners 3-P (T-Score)*						
ADHD Index	17.20	2.044	14.92	3.662	1.76	0.09
Global Index	86.30	4.692	82.92	8.549	1.12	0.27
DSM-5 Inattention	87.60	2.716	82.15	7.221	2.26	0.04
DSM-5 Hyp/Imp	85.10	6.350	83.15	10.367	0.52	0.61
*ARI*						
Parent-rated	0.97	0.55	0.83	0.53	0.60	0.56
Child-rated	0.70	0.53	0.66	0.39	0.24	0.82
*MEWS*	17.50	6.10	16.54	7.86	0.32	0.75
*WREMB-R Total Score*	23.10	6.95	20.54	4.03	1.11	0.28
*CIS*	23.40	9.69	22.00	6.60	0.41	0.68
*Side effects*	16.80	12.90	12.15	5.97	1.15	0.26
**Cognitive Measures**						
*GNG (PI %)*	37.25	17.01	53.46	19.41	-2.093	.049
CPT						
Omissions %	18.33	9.13	12.05	14.15	1.219	.236
Commissions %	*2.98*	2.02	1.941	3.94	.755	.459
*Simon Task (Simon RT Effect)*	99.87	34.304	67.92	31.37	2.326	.030
*Time Discrimination Task (Total Correct)*	66.17	10.45	79.74	14.37	-2.514	.020
*Macworth Clock Task*						
Omissions %	49.25	13.13	30.00	16.99	2.962	.01
Commissions %	10.56	12.24	2.20	1.72	2.447	.023
*WCST*						
Perseverative Errors	13.50	4.33	14.00	5.12	-0.248	.807
Non-Perservative Errors	9.30	4.45	7.00	3.96	1.310	.204
*NIH Working Memory Task*	19.60	16.37	31.92	9.74	-2.254	.035
*Verbal Fluency (% Correct)*	89.75	10.47	93.28	7.89	-0.924	.366
*Semantic Fluency (% Correct)*	93.58	5.25	95.50	5.57	-0.842	.409
**Cognitive Training**						
Grub Ahoy	21.00	8.10	26.92	12.17	-1.33	0.20
Magic Lens	58.50	14.54	58.85	10.64	-0.07	0.95
Monkey Trouble	23.00	10.60	22.31	14.52	0.13	0.90
Peter’s Printer Panic	102.00	12.52	90.39	17.73	1.76	0.09
Treasure Trunk	62.00	19.18	66.15	17.34	-0.54	0.95
**Medication**	***N***		***N***		**χ**^**2**^**(4)**	***p***
Medication-naïve	1		3		2.54	.64
On-medication	1		0			
On-medication, abstained for assessments	2		5			
Off-medication	3		2			

ADHD-RS, Caregiver-rated ADHD Rating Scale;

ARI, Affective Reactivity Index;

CIS, Columbia Impairment Scale Parent;

Kiddie Schedule for affective disorders and schizophrenia present and lifetime version DSM-5 for ADHD;

MEWS, Mind Excessively Wandering Scale;

SD, Standard Deviation;

SCQ, Social Communication Questionnaire;

SDQ, Social Difficulties Questionnaire;

WASI-II, Wechsler Abbreviated Scale of Intelligence;

WREMB-R, Weekly Parent Ratings of Evening and Morning Behavior-Revised.

### 2.3 Outcome measures

#### 2.3.1 Offline cognitive measures

The adult version of the Maudsley Attention and Response Suppression (MARS) Task battery (Westwood et al., 2021) was used to measure motor response inhibition (Go/No-Go Task; dependent variable [DV]: % probability of inhibition [PI]), sustained attention (Continuous Performance Task [CPT]; DV: omission and commission errors), interference inhibition (Simon Task; DV: Simon reaction time effect), and time discrimination (Time Discrimination Task; DV: percentage correct). Other tasks measured vigilance (The Mackworth Clock Task (Lichstein et al., 2000, Mackworth, 1948, PsyTookit, 2017); DV: percentage omissions and commission errors), cognitive flexibility (Wisconsin Card Sorting Task, WCST (PsyTookit., 2018); DV: total and perseverative errors), visuo-spatial WM (C8 Sciences version of the NIH List Sorting Working Memory Task (Tulsky et al., 2014); DV: total score). Given the possibility of a downregulation of left IFC mediated functions, particularly language production, verbal and semantic fluency (DV: percentage correct responses)(Troyer, 2000) were also measured (a full description of tasks, is provided in Westwood et al. (2021)).

#### 2.3.2 ADHD Symptoms and related impairments

Treatment effects in ADHD symptoms was measured with the caregiver-rated ADHD Rating Scale–IV (ADHD-RS) Home Version (DV: Total Scores)(DuPaul et al., 1998) and Conners 3-P (DV: ADHD Index)(Conners, 2008). Also measured were related difficulties and functional impairments (Weekly Parent Ratings of Evening and Morning Behavior-Revised scale, WREMB-R(Wehmeier et al., 2009); Columbia Impairment Scale-Parent version, CIS(Bird et al., 1993)); irritability (child- and caregiver-rated Affective Reactivity Index, ARI(Stringaris et al., 2012)), and mind-wandering (child-rated Mind Excessively Wandering Scale, MEWS)(Mowlem et al., 2019).

#### 2.3.3 Safety measures

Safety was measured with caregiver-rated side effects (Hill and Taylor, 2001) and adverse events (Döpfner and Steinhausen, 2006).

### 2.4 EEG-task description

Participants performed one block of the adult variant of the Maudsley Attention and Response Suppression (MARS) Go/No-Go Task, a measure of motor response inhibition (Penades et al., 2007, Rubia et al., 2007). In 73.4% of trials, a spaceship (Go stimulus) pointing left appeared in the center of the screen and participants had to press the left with their left-index finger arrow key as fast as possible. In 26.6% of trials, a blue planet (No-Go stimulus) appeared in the center of the screen instead of a spaceship and participants had to inhibit their response. Go and No-Go stimuli were displayed for 300 ms followed by a blank screen for 1000 ms. There are 150 trials in total (110 Go trials, 40 No-Go trials). The key dependent measure of the inhibitory performance is the probability of inhibition (PI). For completeness we also report other measures such as premature responses to all trials which is another impulsiveness indicator and the executive go process which includes mean reaction times (MRT), intrasubject response variability (i.e., SD of MRT) and omission errors to go trials. The task duration was 2.5 min. EEG was recorded over 9 min, with a 1-minute gap between rest and task activity, 5-mins resting activity, and ~2.5-mins for task-related activity in the same order for all participants.

### 2.5 EEG system/device

EEG was recorded from an 8-channel DC-coupled recording system using a wearable headset, manufactured by gtec (using Nautilus platform, https://www.gtec.at). Active dry electrodes (Sahara) with gold-plated pins and pre-amplification module were attached to the cap system, which allowed recordings using the 10–20 montage. The EEG data was wirelessly (using Bluetooth) transmitted to the recording laptop.

### 2.6 EEG recording

During all recordings, the pre-amplification module in the active electrodes allows to keep the signal stable (~20 µV) and the impedances below (30 kΩ). Adhesive ground and reference electrodes were positioned at the mastoids. The signal was digitized at a sampling rate of 500 Hz, with additional online filters (bandpass filter, 0.1–100 Hz, and notch filter, 58–62 Hz).

Participants were seated on a height adjustable chair in a testing lab. Stimuli were presented on a laptop at a distance of approximately 30 cm.

### 2.7 EEG pre-processing

Analyses were carried out in the open-source EEGLAB software(Delorme and Makeig, 2004). Researchers were blind to group status during EEG pre-processing, analysis and discussion. The raw EEG data were re-referenced offline to the average reference and were down-sampled to 256 Hz. The raw data were also digitally filtered using basic Finite Impulse Response (FIR) filters between 0.1 Hz and 30 Hz. Prior to re-referencing, flat channels and channels with extremely large artifacts were removed and replaced with topographic spline interpolation. On average, 2 channels were interpolated across all datasets. Sections of data exceeding 200μV were automatically removed. Ocular artifacts were removed using the independent component analysis (ICA) algorithm, runica,(Jung et al., 2000). All other components were back-projected for further analysis. Following the back-projection, all datasets were also visually inspected and sections of data containing residual artifacts were removed manually. All analyses included EEG recordings which had 25% or less data removed, with 150–210 epochs of artifact free data on average (~60 epochs for task performance, ~150 epochs for rest episodes).

### 2.8 QEEG

Quantitative EEG was investigated for resting state and EEG-Go/No-Go Task. Data were segmented into 2 s epochs and power spectra were computed using a fast Fourier transform with a 10% Hanning window. Analyses focused on alpha (8–14 Hz), theta (3–7 Hz) and beta (15–30 Hz) band differences between two groups (anodal vs sham tDCS). EEG absolute power density (μV^2^/Hz) within each frequency band was computed for each electrode individually separately for the task and the rest periods as well as averaged across all electrode sites (Cz, F7, F3, F4, F8, Fpz, Fz, Pz) to reduce the number of comparisons. Due to the main focus being on the electrode over the stimulation site (F8), analyses of the other electrodes are reported in the Supplementary material (Supplementary Analysis 1). To further explore treatment-related change, theta activity was also calculated by subtracting theta activity at post-treatment from EEG activity during baseline (Supplementary Analysis 3).

### 2.9 Statistical analysis

Normality of data was assessed using the Shapiro–Wilk statistic and visual inspection of score distribution. Log 10 transformation of the EEG data and error data was performed to normalize both the EEG and the error data. Furthermore, exploratory pairwise correlational analysis among age, ADHD severity, all EEG and cognitive performance measures were performed and reported in the Supplementary Material. The correlational results indicated that younger age at entry was moderately associated with higher theta and alpha activity during rest at baseline and during task performance at post-treatment, as well as with greater intra-subject variability, slower reaction time and more premature errors during the QEEG Go/No-Go at both baseline and post-treatment (Supplementary Table 2). Greater ADHD severity was also moderately and significantly associated with poorer Go/No-Go PI. There were no other significant correlations (Supplementary Table 2).

Group differences on all outcome measures were tested with repeated measures analysis of covariance (ANCOVA) with Group (anodal vs sham tDCS) as a between-subjects factor and Time (baseline vs post-treatment) as a within-subjects factor, while covarying for baseline age in months and ADHD-RS scores. The covariates were selected to adjust for baseline differences, with the anodal tDCS group being significantly younger and reported higher ADHD-RS Total Score (see Table 1).

The alpha level was set at 0.05. To correct for multiple testing, False Discovery Rate (FDR) correction with Benjamini-Hochberg (Benjamini and Hochberg, 1995) was applied to outcomes with *p*-values less than 0.1, which was applied separately to the different frequency bands, secondary clinical outcomes, and secondary cognitive outcomes. We did not correct for multiple testing on Offline Go/No-Go, or ADHD -RS as these were considered primary outcome measures (see also Westwood et al., 2021)). In the Results section below, we report significant *p*-values before and after FDR correction (hereafter referred to as unadjusted and FDR adjusted, respectively). Analyses were conducted with IBM SPSS Statistics 26 (IBM Corp., Armonk, N.Y., USA).

### 2.10 Ethics

This trial received local research ethics committee approval (REC ID: 17/LO/0983) and was conducted in accordance with the Declaration of Helsinki (Moher et al., 2012).

## 3 Results

Out of the 29 individuals, 6 individuals were excluded from all analyses due to extreme values driven by large EEG artifacts. Only 23 recordings were available for the analysis (n = 10 active, n = 13 sham).

### 3.1 Baseline comparisons

Compared to sham, the anodal tDCS group was on average 2-years younger, had higher ADHD-RS Total & Inattentive Scores and Conners’ 3-P DSM-Inattentive Scores, and fewer years in education. Cognitively, the anodal compared to the sham tDCS group showed significantly poorer performance on the following offline tasks: Go/No-Go (PI%), Simon (Simon RT Effect), Macworth Clock (Omissions & Commissions), and NIH WM Tasks (Total Score) (see Table 1).

### 3.2 EEG outcomes measures

#### 3.2.1 During rest

There was no significant Group-by-Time interaction and no significant main effects of Group or Time on EEG activity (alpha, beta, theta) during rest based on the average of all electrodes or at F8 only (Table 2).

**Table 2:** Summary of adjusted average performance on EEG outcomes, primary and secondary cognitive and clinical outcome measures after sham and anodal tDCS combined with CT. Benjamini-Hochberg adjusted p-values given parentheses.

	Baseline					Post-treatment					ANCOVA					
	Anodal tDCS N = 10		Sham tDCS N = 13			Anodal tDCS N = 10		Sham tDCS N = 13			Time		Group		Time by Group	
	M*	SD	M*	SD	*d*	M*	SD	M*	SD	*d*	F (1,19)	p * *	F (1,19)	p * *	F (1,19)	p * *
**EEG Outcomes**																
*Rest*																
Alpha	0.85	0.44	0.74	0.38	0.26	0.87	0.48	0.80	0.41	0.16	0.27	.61	0.69	.42	0.06	.81
Beta	0.88	0.52	0.72	0.45	0.32	0.88	0.61	0.73	0.52	0.26	0.01	.95	1.31	.27	0.01	.92
Theta	1.24	0.36	1.07	0.31	*0.50*	1.25	0.60	1.07	0.52	0.32	0.84	.37	2.27	.15	0.01	.96
*GNG Task*																
Alpha	0.92	0.58	0.73	0.52	0.34	0.88	0.47	0.75	0.42	0.29	5.26	.03(0.31)	1.43	.25	0.14	.71
Beta	0.96	0.60	0.76	0.54	0.35	0.95	0.59	0.73	0.53	0.39	3.77	.07(0.51)	1.82	.19	0.02	.89
Theta	1.28	0.55	1.12	0.49	0.31	1.25	0.53	1.07	0.48	0.36	4.53	.05(0.43)	1.62	.22	0.44	.52
*Rest F8*																
Alpha	0.85	0.35	0.70	0.35	0.43	0.82	0.42	0.86	0.41	0.10	0.07	.80	0.19	.67	0.59	.45
Beta	0.91	0.45	0.69	0.44	0.49	0.90	0.51	0.71	0.50	0.38	0.46	.83	1.48	.24	0.02	.89
Theta	1.21	0.27	1.05	0.26	*0.60*	1.22	0.48	1.13	0.47	0.19	0.38	.54	1.17	.29	0.07	.80
*Task F8*																
Alpha	0.92	0.42	0.68	0.43	*0.56*	0.87	0.45	0.76	0.46	0.24	6.07	.02(0.31)	1.33	.26	0.04	.85
Beta	0.98	0.48	0.77	0.50	0.43	0.97	0.55	0.71	0.57	0.46	5.61	.03(0.31)	1.28	.27	0.30	.59
Theta	1.26	0.36	1.08	0.37	0.49	1.25	0.45	1.18	0.47	0.15	7.14	.02(0.31)	0.74	.40	0.23	.64
***GNG-EEG Performance***																
*†PI %*	45.05	31.69	48.59	30.02	0.11	45.60	21.27	54.91	20.15	0.45	5.41	.03	0.01	.94	0.36	.56
*RTV (ms)*	124.53	53.13	102.28	50.30	0.43	131.51	35.53	110.68	33.76	*0.60*	0.01	.93	3.56	.08	0.01	.92
*MRT (ms)*	302.68	67.19	291.36	63.64	0.17	325.30	65.08	335.12	61.62	0.15	0.26	.62	0.02	.97	1.39	.26
*Omissions (%)*	0.70	0.70	0.53	0.54	0.27	0.46	1.00	0.47	0.77	0.01	1.58	.23	0.16	.70	0.51	.49
*Premature Errors (%)*	10.73	14.64	9.80	13.86	0.07	8.56	10.09	4.75	9.54	0.39	2.56	.13	0.42	.52	1.34	.26
**Offline Cognitive Outcomes**																
*GNG Task PI (%)*†	40.41	19.33	51.03	18.90	.58	46.07	18.90	51.97	18.57	.33	0.45	.51	1.17	.29	.41	.53
*CPT*†																
Omission (%)	17.45	13.31	12.73	12.10	.40	12.95	10.61	11.83	10.43	.11	2.81	.11	.39	.54	.76	.39
Commission (%)	2.42	3.34	2.373	3.28	.01	2.22	1.63	.95	1.60	.82	1.18	.29	1.08	.31	1.08	.31
*Simon Task (Simon RT Effect)*	97.99	36.52	69.37	35.89	.83	79.711	36.71	44.28	36.06	1.02	0.19	.70	0.13	.72	0.13	.72
*Time Discrimination Task (Total Correct %)*	67.59	14.39	78.65	14.13	.81	62.670	14.82	71.41	14.56	.62	0.32	.58	3.26	.09 (0.13)	0.11	.74
*Mackworth Clock Task*																
Commissions (%)	10.09	9.11	2.56	8.96	.87	6.09	4.75	2.88	4.66	.72	0.68	.42	3.15	.09 (0.11)	3.69	.07 (0.16)
Omissions (%)	47.50	15.47	31.35	15.20	1.01	40.91	15.74	28.34	15.47	.84	0.09	.77	6.17	.02 (0.07)	0.22	.65
*WCST*																
Non-Perseverative Errors	8.58	4.57	7.55	4.49	.24	9.11	4.68	4.07	4.60	1.14	0.15	.70	2.9	.10 (0.10)	3.78	.07 (0.12)
Perservative Errors	12.75	4.90	14.58	4.81	.39	12.74	5.14	11.36	5.05	.28	6.79	.02 (0.14)	0.01	.91	1.76	.20
*NIH WM Task (Total Score)*	19.13	14.72	32.29	14.46	.95	25.88	19.42	35.17	19.08	.51	2.20	.15	2.77	.11	0.29	.60
*Verbal Fluency (% Correct)*	89.70	9.46	93.32	9.29	.40	92.93	4.71	98.03	4.63	1.15	2.78	.11	2.56	.13	0.15	.70
*Semantic Fluency (% Correct)*	93.89	5.69	95.26	5.59	.25	96.25	2.71	97.85	2.66	.62	0.98	.33	0.90	.35	0.01	.93
**Clinical Outcomes**																
*ADHD-RS*†																
Total Score‡	43.13	6.94	39.06	6.86	.62	29.42	8.10	26.22	8.00	.42	0.01	.92	2.21	.15	0.04	.85
Inattention	23.18	2.45	22.56	2.41	.27	16.29	4.86	14.47	4.78	.40	3.67	.07	0.78	.39	0.36	.56
Hyp/Imp	17.65	2.45	18.27	2.41	.27	12.85	5.16	11.96	5.07	.18	8.40	.01	0.01	.93	0.54	.47
*Conners 3-P ADHD Index*	16.11	2.71	15.76	2.66	.13	12.39	4.33	8.01	4.26	1.07	0.27	.61	4.19	.06 (0.09)	3.25	.09 (0.09)
*ARI*																
Parent	.89	.57	.897	.56	.02	.858	.53	.50	.52	.72	0.02	.88	0.72	.41	1.99	.18
Child	.69	.52	.662	.51	.06	.637	.54	.45	.53	.38	0.26	.62	0.26	.63	1.25	.28
*MEWS*	18.01	8.00	16.15	7.86	.25	17.66	9.95	15.95	9.78	.18	0.08	.78	0.23	.64	.003	.96
*WREMB-R*	21.49	4.77	21.78	4.69	.06	15.77	6.83	15.64	6.71	.02	0.43	.52	0.001	.97	0.02	.89
*CIS*	21.20	7.5	23.70	7.37	.35	14.88	9.43	17.63	9.17	.31	5.60	.03 (0.09)	0.58	.46	0.01	.94
**Safety†**																
*Side Effects*	16.07	10.57	12.72	10.39	.33	12.71	7.14	10.99	7.02	.25	0.94	.34	0.52	.48	0.18	.68
*Adverse Effects*	–	–	15.72	1.88		–	–	15.68	1.84	.02	0.002	.96	–	–	–	–

ADHD-RS, ADHD Rating Scale;

ARI, Affective Reactivity Index;

CIS, Columbia Impairment Scale-Parent;

GNG, Cog/No-Go

MEWS, Mind Excessively Wandering Scale;

MRT, Mean Reaction Times;

PI, Probability of Inhibition;

RTV, intra-subject response time variability

SD, Standard Deviation;

WREMB-R, Weekly Parent Ratings of Evening and Morning Behavior-Revised

* , Adjusted values as predicted by the repeated-measures ANOVA, adjusting for ADHD-RS Total Score and age at entry. * *, Benjamini-Hochberg adjustment was applied to significant p-values only. †Benjamini

Hochberg adjustment was not applied to these measures. ‡, covaried for age at entry in months only. Cohen’s d: d< 0.30 - small effect size, d≥ 0.50 – medium effect size, d≥ 0.80 -large effect size.

#### 3.2.2 During go/no-go task performance

Based on the average across all electrodes, there was no significant Group-by-Time interaction effect and no significant main effect of Group on EEG activity during Go/No-Go Task performance. There was a main effect of Time on theta and alpha activity, with lower theta and alpha activity at post-treatment compared to baseline, but this effect did not survive FDR correction (Table 2). There was a significant time-by-age interaction (theta, F(1,19) = 6.72, unadjusted p = 0.018, FDR adjusted p = 0.31; alpha, F(1,19) = 5.42,unadjusted p = 0.032, FDR adjusted p = 0.31) showing higher theta and alpha activity in younger subjects at pre- compared to post-treatment, but this interaction was no longer significant after FDR correction.

Based on F8 only, there was no significant Group-by-Time interaction effect and no significant main effect of group on EEG activity (alpha, beta, theta). There was a significant main effect of Time, showing lower alpha, beta and theta activity at post-treatment compared to baseline, but this was no longer significant after FDR correction. There was also a significant time-by-age interaction effect (theta, F(1,19) = 9.91,unadjusted p = 0.006, FDR adjusted p = 0.10; alpha, F(1,19) = 6.39,unadjusted p = 0.021, FDR adjusted p = 0.31; beta (F(1,19) = 5.86, unadjusted p = 0.026, FDR adjusted p = 0.31), showing higher theta and alpha activity in younger subjects at pre- compared to post-treatment, which was no longer significant after FDR correction (Table 2).

### 3.3 Other clinical & offline cognitive outcome measure

There was no significant main effects of Group, Time, or Group-by-Time interaction effect that survived FDR Correction on clinical or offline cognitive measures (Table 2), except a significant main effect of Time in ADHD-RS Hyperactivity/Impulsivity Subscale (F(1,19) = 8.4, *p* = 0.01).

## 4 Discussion

This is the first double-blind, sham-controlled RCT to test the neurofunctional mechanisms of action of multi-session, anodal tDCS over the rIFC combined with cognitive training in children and adolescents with ADHD on QEEG spectral power. There were no significant differences between sham and anodal tDCS on QEEG spectral power during rest and Go/No-Go Task performance. Further, both sham and real tDCS showed lower EEG spectral power at post-treatment compared to baseline, although this did not survive FDR correction for multiple comparisons. Finally, there were no statistical differences in these subgroups in clinical and cognitive outcome measures. These null findings in QEEG spectral power, and the pattern of results in clinical and cognitive outcomes in this subgroup of 23 children and adolescents with ADHD extends the evidence of overall null findings in clinical or cognitive outcomes following anodal tDCS over the rIFC relative to sham in the original sample of 50 children and adolescents with ADHD published previously (Westwood et al., 2021). The lack of tDCS-related electrophysiological effects as measured in EEG measures during rest and a cognitive control task, may underlie the lack of clinical and cognitive benefits of tDCS. These null findings are furthermore complemented by the lack of a significant correlation between the QEEG and Go/No-Go cognitive performance measures in the same subgroups. However, the null EEG findings need to be considered with caution considering the small, underpowered sample.

The lack of a significant tDCS effect on QEEG measures contrasts with previous evidence of reduced absolute theta power during rest after a single session of anodal compared to sham tDCS over the rIFC in adults without ADHD (Jacobson et al., 2012). Another study in 15 adolescents with ADHD found enhanced ERP amplitudes (e.g., N2 and P3)(Breitling et al., 2020; Dubreuil-Vall et al., 2020) during an n-back WM task after a single-session of conventional anodal tDCS or HD-tDCS over rIFC. Nevertheless, our findings support studies that used more fine-grained measures of EEG activity and failed to show differences between anodal and sham tDCS over the left DLPFC in EEG connectivity during rest in 50 adults with ADHD(Cosmo et al., 2015), or any effect on both rest- and task-based EEG power spectrum following anodal tDCS in neurotypical adults (Hill et al., 2019, Holgado et al., 2019, Miller et al., 2015). Together, our findings extend a quantitative review showing little-to-no reliable neurophysiological effects of anodal tDCS in neurotypical adults (Horvath et al., 2015a).

These findings might suggest that multi-session tDCS over rIFC combined with CT had no effect on EEG measures of spectral power during rest or task performance in children with ADHD. However, this lack of effect on EEG spectral power could be related to the underpowered sample size (n = 23) and/or baseline differences, with the anodal tDCS group being younger and having higher parent ratings on ADHD-RS and Conners’ 3-P compared to sham. A potential confounding effect of age chimes with previous evidence indicating that QEEG varies as a function of age (Snyder and Hall, 2006). Children with ADHD consistently show excessively high absolute delta and theta power compared to adults with ADHD, and this excessive increase in slow frequencies decreases with age and hence becomes less different from neurotypical individuals (Kiiski et al., 2020, McVoy et al., 2019). Furthermore, with the original sample of 50 children with ADHD, older but not younger participants showed less improvement in ADHD symptoms in the anodal versus sham tDCS group at post-treatment (Westwood et al., 2021). This finding might be explained by less ADHD severity in the older compared to the younger participants at baseline. Further, it is important to note that by covarying for baseline differences in age and ADHD-RS scores we expended even further our limited statistical power. Thus, adequately powered studies should investigate how age affects EEG activity following anodal tDCS in individuals with and without ADHD.

Both groups showed improvement in EEG Go/No-Go PI and offline WCST Perseverative Errors from baseline to post-treatment. A similar effect of time effect was observed in the larger sample of 50 ADHD children for cognitive performance on an offline Go/No-Go and other offline tasks measuring ADHD-related EF (e.g., Simon RT Effect; Verbal Fluency)(Westwood et al., 2021). However, in the absence of a Time-by-Group interaction, the baseline to post-treatment improvement in QEEG Go/No-Go PI and WCST Perseverative Errors could either be due to CT, to placebo and/or to practice effects. The correlational analysis (reported in the Supplementary material. Supplementary Analysis 3) showed a negative correlation between EEG Go/No-Go PI with ADHD severity (ADHD-RS total), but not with CT and EEG measures, suggesting the improvement in EEG Go/No-Go PI was not related to CT or EEG changes. However, we cannot rule out that the lack of correlation is related to a lack of power, given the small sample of participants analysed. Nevertheless, findings from the EEG Go/No-Go Task and offline cognitive tasks broadly replicate cognitive findings in the whole group (Westwood et al., 2021).

Given the insufficient EEG data at the 6-month follow-up assessment, we could not test for potential longer-term tDCS effects on EEG measures after a period of consolidation (Martin et al., 2014). However, thus far, tDCS studies in ADHD that show longer-term clinical and/or cognitive effects have all reported significant effects at post-treatment that persisted only in the order of weeks, not months (Cachoeira et al., 2017, Soff et al., 2017). This study found no post-treatment effects, and the analysis of the whole group found no clinical or cognitive effects at follow-up (Westwood et al., 2021), making longer-term effects in EEG measures unlikely. Future studies should investigate consolidation effects in EEG measures following anodal tDCS in children with ADHD, which – at the time of writing – has not been studied. Other neurotherapies, such as EEG neurofeedback (NF) or fMRI-NF(Alegria et al., 2017), have shown stronger clinical effects at follow-up than at post-treatment, indicating a delayed consolidation of neuromodulatory effects, suggesting neuroplasticity (Aggensteiner et al., 2019, Alegria et al., 2017, Enriquez-Geppert et al., 2019). Unfortunately, we only collected EEG data of 16 participants at follow-up, and it was hence not possible to investigate the longer-term effects of anodal tDCS on EEG activity in the current study. Future studies should therefore focus on evaluating the effect of tDCS on neural activity at multiple follow-up points (e.g., 3 and 9 months) and using better spatially resolved techniques such as fMRI.

### 4.1 Limitations

Although this RCT had a relatively larger sample (n = 50) than other tDCS studies in children and adolescents with ADHD, EEG data could only be collected for 29 participants at post-treatment (of which only n = 23 were analysed), and there was insufficient EEG data at follow-up (n = 16) to test longer-term effects of tDCS. Thus, this sample of 23 participants is only sufficiently powered to detect exceptionally larger effects (e.g., *d >* 1.00), which could be related to the statistical null findings. These findings should therefore be replicated using adequately powered sample sizes. The attrition rate of this study was very high (42%) compared to studies using QEEG in children above the age of 4 either with and without ADHD (5%−25%)(Aldemir et al., 2018; Bell and Cuevas, 2012; Skirrow et al., 2015), which is likely due to the discomfort of the dry EEG electrodes (Lim et al., 2019) that were chosen for their ease of application. Future studies should aim at choosing electrodes, which induce the minimum possible discomfort in children and adolescents with ADHD. Technological advances mean that dry active EEG electrode can reliably estimate EEG spectral power and ERP components, but they still suffer high interelectrode impedance and therefore very high noise levels (Mathewson et al., 2017), as evidenced in our data and in the exclusion of 6 participants as outliers. Future studies with dry electrodes should consider including tasks with longer duration (15 min) and a sufficient number of trials (100 or more trials), which should boost statistical power and provide more confidence estimates of tDCS effects. Additionally, this study did not include EEG triggers to capture the stimuli presentation and had only eight electrodes, resulting in a limited choice of analyses. Therefore, we cannot exclude the possibility that event-related analyses might have been more informative and led to positive findings. Future studies should take advantage of both the time and frequency domain of EEG, and thus perform various event-related time-frequency analyses. The use of 64 or more electrodes would also allow for EEG source-level analysis, which could be invaluable in understanding the exact cortical origin of the EEG signal.

### 4.2 Conclusions

This study in 23 children with ADHD failed to show a differential effect of 15 sessions of anodal versus sham tDCS & CT on EEG spectral power, and cognitive or clinical outcomes. The findings extend our previous findings in a larger group of 50 ADHD children of no superior effects of anodal versus sham tDCS & CT on clinical or cognitive measures by showing no underlying neurofunctional mechanism of action in a subgroup (Westwood et al., 2021). Although tDCS is becoming increasingly accepted into clinical practice and viewed as an alternative to medication by parents (Buchanan et al., 2020, Sierawska et al., 2021, Sierawska et al., 2019), our findings suggest that rIFC stimulation may not be indicated as a treatment choice for neurophysiological, cognitive or clinical remediation for children and adolescents with ADHD. Larger RCTs need to be conducted to explore different protocols (such as different stimulation sites, amplitude, frequency, etc) titrated to the individual and using cognitive, clinical, and neural outcome measures to comprehensively assess the effect of tDCS and its underlying mechanisms of action on brain activity in ADHD.

## Funding

This work was supported by grants from 10.13039/501100000317Action Medical Research (GN2426), the 10.13039/100013999Garfield Weston Foundation, and 10.13039/501100000272National Institute for Health Research (NIHR) Biomedical Research Center at South London and the Maudsley NHS Foundation Trust, and 10.13039/501100000764King's College London to KR. KR has received additional research support for other projects from the 10.13039/501100000265Medical Research Council (MR/P012647/1), and the National Institute for Health Research (NIHR) Biomedical Research Center at South London and the Maudsley NHS Foundation Trust, and King's College London. The views expressed are those of the author(s) and not necessarily those of the NHS, the NIHR or the Department of Health and Social Care. The funders were not involved in the collection, analysis and interpretation of data; in the writing of the report; or in the decision to submit the article for publication.

## CRediT authorship contribution statement

Contributions according to Contributor Roles Taxonomy (CRediT) (casrai.org/credit/). Conceptualization. Katya Rubia, Roi Cohen-Kadosh, Samuel J. Westwood, Natali. Bozhilova, Marion Criaud, Sheut-Ling Lam, Steve Lukito, Bruce Wexler, Philip Asherson. Data curation. Samuel J. Westwood, Natali Bozhilova, Marion Criaud, Sheut-Ling Lam, Steve Lukito, Sophie Wallace-Hanlon and Afroditi Kostara. Formal analysis. Samuel J. Westwood, Natali Bozhilova, Roi Cohen-Kadosh, and Katya Rubia, Funding acquisition. Katya Rubia, Bruce Wexler, Roi Cohen-Kadosh, Philip Asherson. Investigation, Samuel J. Westwood, Marion Criaud, Sheut-Ling Lam, Steve Lukito, Sophie Wallace-Hanlon, Olivia S. Kowalczyk, Afroditi Kostara and Joseph Mathew. Methodology. Katya Rubia, Roi Cohen-Kadosh, Bruce Wexler, Samuel J. Westwood, Marion Criaud, Sheut-Ling Lam, Steve Lukito, Olivia S. Kowalczyk, Philip Asherson. Project administration. Samuel J. Westwood and Katya Rubia. Resources. Katya Rubia, Bruce Wexler, Roi Cohen-Kadosh, Philip Asherson. Software. Bruce Wexler, Katya Rubia, Marion Criaud,. Supervision. Samuel J. Westwood and Katya Rubia. Validation. Samuel J. Westwood and Katya Rubia. Visualization. Natali Bozhilova. Writing – original draft preparation. Samuel J. Westwood, Natali Bozhilova, and Katya Rubia. Writing – review and editing. Samuel J. Westwood, Natali Bozhilova, Katya Rubia, Marion Criaud, Sheut-Ling Lam, Steve. Lukito, Sophie Wallace-Hanlon, Olivia S. Kowalczyk, Bruce Wexler, Roi Cohen-Kadosh.

## Compliance with ethical standards

The authors assert that all procedures contributing to this work comply with the ethical standards of the relevant national and institutional committees on human experimentation and with the Helsinki Declaration of 1975, as revised in 2008. This trial received research ethics committee approval from The NHS Research Authority, London Camberwell St Giles Research Ethics Committee (REC ID: 17/LO/0983) in November 2017.

## Informed Consent Statement

Informed consent was obtained from all participants involved in the study.

## Conflicts of Interest

BEW is Chief Scientist and an equity holder in the Yale Start Up company C8 Sciences that sells the cognitive training program evaluated in this study. RCK serves on the scientific advisory boards for Neuroelectrics and Innosphere. PA reports honoraria for consultancy to Shire/Takeda, Eli Lilly and Novartis; educational and research awards from Shire, Lilly, Novartis, Vifor Pharma, GW Pharma and QbTech; and speaking at sponsored events for Shire, Lilly, Flynn Pharma and Novartis. KR has received funding from Takeda pharmaceuticals for another project and consultancy fees from Lundbeck and Supernus which were paid into King’s College London and used for research.

## Data Availability

Data is available upon reasonable request.
